# Geographic Variation in Top-10 Prescribed Medicines and Potentially Inappropriate Medication in Portugal: An Ecological Study of 2.2 Million Older Adults

**DOI:** 10.3390/ijerph191912938

**Published:** 2022-10-10

**Authors:** Vânia Rocha, Ana Isabel Plácido, Daniela A. Rodrigues, Ana Barbara Tavares, Adolfo Figueiras, Fátima Roque, Maria Teresa Herdeiro

**Affiliations:** 1 Department of Medical Sciences, Institute of Biomedicine (iBiMED), University of Aveiro, 3810-193 Aveiro, Portugal; 2 Research Unit for Inland Development, Polytechnic of Guarda (UDI-IPG), 6300-559 Guarda, Portugal; 3 Department of Preventive Medicine and Public Health, University of Santiago de Compostela, 15702 Santiago de Compostela, Spain; 4 Health Research Institute of Santiago de Compostela (IDIS), 15706 Santiago de Compostela, Spain; 5 Consortium for Biomedical Research in Epidemiology and Public Health (PCIBER Epidemiology and Public Health-CIBERESP), 28001 Madrid, Spain; 6 Health Sciences Research Centre, University of Beira Interior (CICS-UBI), 6200-506 Covilhã, Portugal; 7 Sociedade Portuguesa de Farmacêuticos dos Cuidados de Saúde (SPFCS), Rua D. Manuel I, 74 1º Piso, 3030-320 Coimbra, Portugal

**Keywords:** older adults, prescription drugs, potentially inappropriate medication, geographic variation

## Abstract

Multiple medication intake by older adults is considered a serious public health concern since it is associated with increased risk of adverse drug reactions and potentially inappropriate medication (PIM). This study described the top-10 prescribed active substances considering geographical distribution and PIM prescription in older adults. A cross-sectional ecological study using data on the active substances prescribed to people aged 65 years or older during 2020 was conducted. Information on active substances and the respective defined daily doses (DDD) stratified by age group, sex and region were collected from a Portuguese health administrative database. The average number of prescribed packages and DDD per 1000 inhabitants per day of top-10 active substances were assessed. This study included a total of 2,228,090 older adults (58% females). The furosemide and atorvastatin were the active substances with higher prescription rates (mean DDD/1000 inhabitants/day) in all ARS in both males and females, in comparison with the other top-10 active substances. Our results showed geographic differences in prescription, illustrated by more prescriptions in ARS North and Centre and fewer prescriptions in ARS Algarve. In females, two out of the 10 most prescribed active substances were PIM (benzodiazepines and opioids). Geographic disparities in PIM prescription across Portuguese regions were also observed. This study shows that drugs for the cardiovascular system were the active substances most prescribed to older adults. The prescription of benzodiazepines and opioids, classified as PIM, among females, alerts officials to the need of health policies to decrease inappropriate medication. The observed geographic differences in the 10 most prescribed active substances and in PIM prescription emphasized the importance of investing in medication optimization across the Portuguese regions.

## 1. Introduction

The worldwide population is ageing fast: by 2050, 1 out of 6 people will be over the age of 65 [1]. Portugal is among the top five countries with the highest percentage of older adults, showing an increase from 9.4% in 1970 to 22.3% in 2020 [2]. As life expectancy increases, the prevalence of multimorbidity [3,4] and medication use [5] also rises. The use of multiple medications in older adults is considered a public health concern since it is commonly associated with a higher risk of adverse drug reactions due to the metabolic changes and decreases in drug clearance associated with ageing [5,6]. Moreover, older adults have been commonly excluded from clinical trials, leading to a lack of evidence regarding the safety and efficacy of many used medications in this population [5,7,8,9].

Medications are considered inappropriate when the risks of their use are greater than their potential benefits or when they are prescribed in inappropriate doses and/or for unsuitable durations [10,11]. Prior studies [12,13,14] reported that in older adults, the potentially inappropriate medication (PIM) prevalence ranges from 21% to 79% according to the different health care contexts. Inappropriate drug treatment has also been associated with various adverse clinical outcomes, for instance frailty, falls, renal failure, poor physical function, impaired health-related quality of life [5,7,8,9], higher risk of hospitalization, and mortality [12,13,14]. Thus, PIM leads to negative health consequences and substantial financial costs for individuals and society [5,14,15]. 

Literature also suggests an increasing concern regarding the geographic variation in health care utilisation and costs [16,17]; although in Portugal, medication cost accounts for 14.8% of all health spending [18], few studies have addressed the geographic differences in medication prescription in older adults yet [16,19], in particular the PIM prescription [20].

The optimisation of medication prescription and improvements in therapeutic effectiveness across regions are therefore a worldwide priority for improving the health and well-being of older adults and consequently reducing health care costs [21]. Additionally, the use of an ecological approach allows for geographical comparisons across regions considering the population as the unit of observation, and this approach is of great importance when individual data are not available [22,23,24]. Thus, this study aimed to describe the top-10 prescribed medications in older adults considering geographical distribution, and PIM prescription in this population.

## 2. Materials and Methods

### 2.1. Study Design and Data Source

A cross-sectional ecological study was conducted on the medications prescribed to people aged 65 years or older from all Portuguese Health Administrative Regions (Administração Regional de Saúde—ARS). Data on the prescribed medication from 1 January–31 December 2020 were retrieved between 1 September and 31 November 2021 from the official System of Information and Monitoring of the Portuguese NHS (SIM@SNS) public-access platform [25], which was developed by the shared services of the Health Ministry (Serviços Partilhados do Ministério da Saúde, SPMS). Data retrieved included active substances and defined daily doses (DDD) by age group, sex and ARS. 

All legal residents of Portugal, including non-working residents, are entitled to free access to the Portuguese National Health Service (NHS). The Portuguese NHS is predominantly financed by taxes and some out-of-pocket payments that include co-payment for a wide range of services, though there are income-based exemptions for certain population groups. For instance, older adults with an average salary 1.5 times below the social support index are exempt from co-payment for any publicly provided services. To promote transparency and facilitate data sharing, the Portuguese NHS created the public database used in this study. This database reports prescription data of public primary care systems. The data includes the number of packages, DDD, and the number of generic of each active substance stratified by sex, age, and region. 

Data on active substance prescriptions were only obtained from outpatient care facilities in the public health sector and not from the private sector. All the information reported in the database is anonymized and aggregated by health care service, age group, and sex. Considering that the Portuguese health care service covers all Portuguese residents and the fact that the database reported data on all public health care services, this database is a valuable and trusted and valuable source of information.

### 2.2. Setting

Portugal has a population of around 10.3 million inhabitants with about 2.3 million older adults (≥65 years old) [26].

The Portuguese mainland ARS are divided into five areas: North, Centre, Lisbon, and Tagus Valley, Alentejo, and Algarve. Some additional information on the five ARS is provided in the Table S1 (in Supplementary Materials).

### 2.3. Medication

The list of 10 most prescribed active substances was extracted from the SIM@SNS platform and classified using the corresponding Anatomical Therapeutic Classification (ATC) code [27]. Each active substance was considered PIM if listed on the European Union (7)-PIM list [28] adapted for the Portuguese population [29]. Nevertheless, it was not possible to apply all the EU (7) PIM List criteria, due to the information available in the database. Therefore, the following drugs were excluded from the analysis: (a) drugs in which the classification of PIM was dependent on dose and/or duration of treatment and/or therapeutic scheme; (b) drugs classified as PIM that have lost their marketing holder in Portugal or that are not currently being marketed; (c) and PIM without a defined daily dose (DDD). 

The active substances consumption was quantified in terms of number of prescribed packages and DDD per 1000 inhabitants per day. The concept of DDD was developed as a standard measure of drug utilisation which represents the average maintenance dose per day of a drug when used for its major indication in adults [27,30]. The DDD is a technical unit of comparison and overcomes difficulties in comparing prescriptions of different price, pack size, duration, and dose [27,30]. The DDD per 1000 inhabitants per day also allowed us to compare between the different ARS, providing an approximate estimate of the proportion of the study population treated daily with a particular drug or group of drugs [31].

### 2.4. Data Analysis

Descriptive statistics using absolute and relative frequencies were performed to characterise the sample (age, sex, and region). The average number of prescribed packages and DDDs per 1000 inhabitants per day of top-10 level 5 ATCs were calculated using mean and standard deviation (SD), and the results are illustrated with column charts. The study population used to calculate both estimates per 1000 inhabitants per day was the Portuguese population by administrative regions as illustrated in Table S2 (Supplementary Materials).

## 3. Results

### 3.1. Sociodemographic Data

A total of 2,228,090 older adults from the five Portuguese regions were analysed: 58% were females, and about 51% were aged from 65 to 74 years; the remaining were 75 years of age or older (Table S3, in Supplementary Materials).

### 3.2. Top-10 Prescribed Active Substances

The list of the 10 most prescribed active substances was ordered by dispensed packages (Table S2, in Supplementary Materials) and corresponded to a median of 14.4% of all prescribed medicines, from a minimum of 1.0% in Algarve to a maximum of 39.1% in Alentejo.

**Figure 1:**
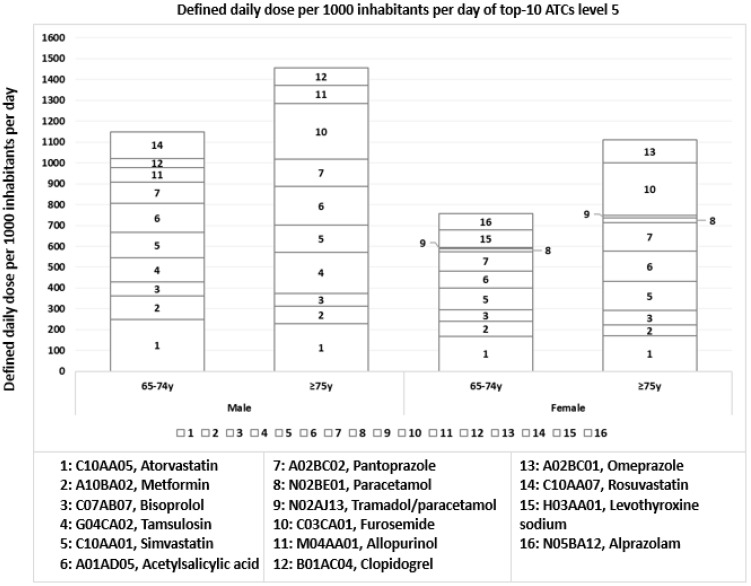
Mean number of defined daily dose per 1000 inhabitants per day of top-10 ATCs level 5, stratified by sex and age in all Portuguese regions.

The most prescribed active substances in both males and females from all age groups were the lipid modifying agents, such as atorvastatin, rosuvastatin, and simvastatin, with mean DDDs of 203.9 ± 40.8, 126.4 and 124.7 ± 40.8 per 1000 inhabitants per day, respectively (Figure 1). Medicines for thyroid therapy (levothyroxine sodium), analgesics (paracetamol and tramadol/paracetamol fixed-dose combination) and benzodiazepines (alprazolam) were mainly found among females, while antigout preparations (allopurinol) were found among males. In the top-10 prescribed active substances, diuretics (furosemide) were mainly prescribed in the age group ≥75 years in both males and females with a mean DDD of 261.0 ± 9.8 per 1000 inhabitants per day (Figure 1). 

### 3.3. Geographic Variation in Prescription

**Figure 2:**
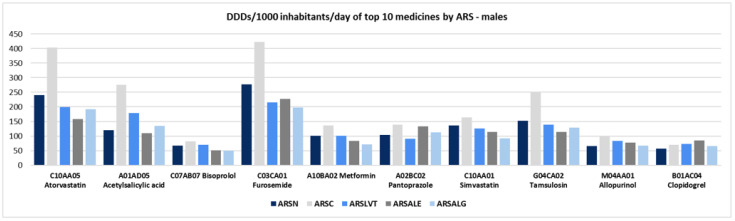
Defined daily dose (DDD) per 1000 inhabitants per day in top-10 level 5 ATCs by health administrative region (ARS) in males. Legend: ARS—North (ARSN), Centre (ARSC), Lisbon-Tejo Valley (ARSLVT), Alentejo (ARSALE), Algarve (ARSALG).

**Figure 3:**
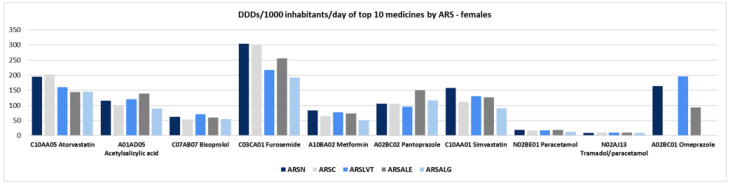
Defined daily dose (DDD) per 1000 inhabitants per day in top-10 level 5 ATCs by health administrative region (ARS) in females. Legend: ARS—North (ARSN), Centre (ARSC), Lisbon-Tejo Valley (ARSLVT), Alentejo (ARSALE), Algarve (ARSALG).

Figure 2 and Figure 3 illustrate the mean DDD per 1000 inhabitants per day in top-10 level 5 ATCs by ARS and stratified by sex. In males, the active substances with the highest mean DDDs per 1000 inhabitants per day were furosemide and atorvastatin. For both substances, higher mean DDDs were found in ARS Centre (furosemide: 422.8; atorvastatin: 403.7 ± 67.7) and ARS North (furosemide: 276.7; atorvastatin: 240.3 ± 10.5) than in the other ARS. For instance, the mean DDD per 1000 inhabitants per day of these substances in ARS Centre was more than twofold higher than the mean prescribed in ARS Algarve (furosemide: 197.4 and atorvastatin: 191.3 ± 1.3) (Figure 2; Table S4).

In females, the active substances with the highest mean DDDs per 1000 inhabitants per day were also furosemide and atorvastatin, with higher mean DDDs found in ARS North (furosemide: 304.4; atorvastatin: 195.0 ± 3.5) and ARS Centre (furosemide: 300.2; atorvastatin: 202.2 ± 0.4) than in the other ARS (Figure 3; Table S5, in Supplementary Materials). Additionally, females were prescribed two proton pump inhibitors (PPI), pantoprazole and omeprazole, while in men, only pantoprazole was prescribed (Figure 2 and Figure 3; Tables S4 and S5, in Supplementary Materials).

### 3.4. PIM Prescription

**Figure 4:**
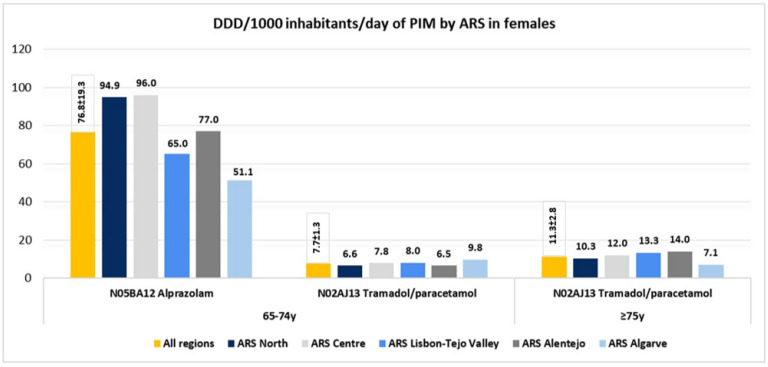
Defined daily dose (DDD) per 1000 inhabitants per day of potentially inappropriate medication (PIM), by health administrative region (ARS). Legend: ARS—North (ARSN), Centre (ARSC), Lisbon-Tejo Valley (ARSLVT), Alentejo (ARSALE), Algarve (ARSALG).

In females, 2 of the 10 most prescribed medications were PIM (Figure 4). Considering all regions, at ages 65 to 74, mean DDDs of 76.8 ± 19.3 of alprazolam and 7.7 ± 1.3 of tramadol/paracetamol per 1000 inhabitants per day were found. At 75 years of age or older, only one PIM was prescribed, tramadol/paracetamol fixed-dose combination, with an increase to 11.3 ± 2.8 DDD/1000 inhabitants/day, in comparison with the mean at ages 65 to 74 (Figure 4). Differences in prescription across regions were observed. Again, ARS Centre showed the highest mean DDD/1000 inhabitants/day for alprazolam (96.0), which was almost twofold higher than in ARS Algarve (51.1). Regarding the tramadol/paracetamol fixed-dose combination at ages 65 to 74, values were similar across regions, and at 75 years of age or older, the highest mean DDD/1000 inhabitants/day was found in ARS Alentejo (14.0), which was twofold higher than in ARS Algarve (7.1) (Figure 4). 

## 4. Discussion

This study described the top-10 active substances prescribed to older adults by age group and sex. The most-prescribed active substances belong to the cardiovascular system, regardless of sex, specifically atorvastatin in individuals from 65 to 74 years old and furosemide in those of 75 years or older. These findings agreed with previous evidence showing that cardiovascular disease is the leading cause of disability and mortality in Portugal and worldwide [32,33,34]. Nevertheless, the modifiable and preventable nature of the main risk factors for cardiovascular disease (i.e., high systolic blood pressure, dietary risks, high cholesterol, tobacco, high body mass index, among others) highlight the need for interventions to address these risk factors, namely, changes in lifestyle together with investments to reduce pharmacological treatments with potential to diminish disease burden and health costs [32,35,36].

The results of our study constitute real-world data about patterns of prescription in the entire older population in mainland regions of Portugal. This information can be important for facilitating the development quality indicators and for promoting the electronic monitoring of prescriptions in Portuguese older adults. 

Geographic differences in prescription and specifically in PIM were found, with higher mean DDDs per 1000 inhabitants per day observed in the Centre and North regions and a lower mean DDD in Algarve. Geographic variation in medicines prescription has been documented in the literature [19,20,37,38,39], but few therapeutic classes have been explored, mainly cardiovascular, psychotropics, opioids, and antibiotics; many therapeutic classes remain unexamined despite the importance of this information for reducing inappropriate prescribing [39]. 

Prior studies addressing geographic differences in PIM reported that higher age and female gender were the main individual characteristics associated with an increased likelihood of PIM [20,40]. In fact, our sample was composed of older adults, and we also found that the PIM prescription was mainly in females, primarily the prescription of benzodiazepines [41] and opioids [42], which fully agreed with this previous evidence [20,40,43,44]. Several factors have been pointed out to explain this trend, namely, the greater demand for health care services among females than males [45], their tendency to live longer and have more physical or psychological health problems, their higher report of chronic conditions including pain [46] or their higher tendency to be prescribed psychotropic medication compared with men [47,48]. 

Other factors related to the regions’ characteristics were also associated with more PIM prescription: lower socioeconomic status, lower access to health services, lower access to general practitioners (GPs), and higher prevalence of chronic diseases [20]. However, in contrast to [20], our findings suggest that Algarve, in which PIM prescription is lower than in the other ARS, is a region characterized by lower pensions from either retirement or disability and lower access to GPs [49,50,51]. We may hypothesize that the lower socioeconomic status due to the lower pensions and the lower access to GPs of individuals from Algarve in 2020 [49,50,52] might explain their lower access to the PIM prescription, being associated with lower purchasing power, as illustrated in Table S1 in Supplementary Materials. Moreover, a previous study also conducted in Portugal showed that the Algarve region presented the lowest values of cardiovascular risk factors, although they had higher levels of smoking [35], which supports our results. Additional studies also reported that the Centre region presented the highest admission and mortality rates for ischemic heart disease [36] and had one of the highest ageing ratios in 2020 [53], which might further explain the higher prescription rates in the Centre region. According to the Portuguese Institute of National Statistics 2020, Alentejo is one of the most aged regions, showing the highest prevalence of chronic diseases (45.5%), followed by the North (44.8%) and Centre (43.3%), also supporting our findings. Nevertheless, more research is required to investigate geographic differences in prescription by regions since they might be associated with over- or undertreatment and may require public health policies to reduce inappropriate prescribing [20,39]. 

PPI prescription in older adults was also found, pantoprazole in males and both pantoprazole and omeprazole in females. Evidence is consistent in showing the associations between longer duration of PPI therapy and numerous adverse effects that are a concern in older adults such as osteoporotic-related fractures, infections, community-acquired pneumonia, vitamin B12 deficiency, kidney disease, and dementia [54,55]. Although in our study we could not assess the inappropriate use of these active substances since we do not have information on the treatment duration, the high prescription of PPI and the presence of geographic differences highlight the need for periodic evaluations of the use of these active substances, considering the importance of investments in treatment discontinuation in this population.

### Strengths and Limitations

The national coverage of the active substances prescribed to the entire Portuguese population of older adults is the strong point of this study, as it provides a prescription profile alerting for the risks of inappropriate medication in this population. Some limitations should also be recognized. The main limitation of our study design is the ecological fallacy since individual data were not available and the study of the Portuguese regions might be subject to aggregation bias. Although these were not available, data on the individuals’ health conditions and socioeconomic indicators would have strengthened our results. Another limitation of this study is associated with the data source, as our data on active substances prescription were only obtained from the outpatient care of the public health sector, and not from the private sector, and thus may not reflect the total prescriptions within the population. Additionally, the nature of the data and the way the data were captured in the SIM@SNS public-access platform only allowed us to perform descriptive statistics. The cross-sectional nature of the study limits the ability to detect the inappropriate use of PPI by the application of all criteria on the EU (7) PIM List. Additional longitudinal studies might help to elucidate this issue. Finally, the cross-sectional design and the data availability also prevented us from assessing the impacts of the COVID-19 pandemic on the medications prescribed for older adults.

## 5. Conclusions

This study found that 4 of the 10 most prescribed active substances to older adults belong to the cardiovascular system group, regardless of sex, suggesting the high prevalence of cardiovascular diseases in this population. The prescription of benzodiazepines and opioids in females, classified as PIM, alerts officials to the need of public health policies to reduce inappropriate medication. Geographic differences in the 10 most prescribed active substances and in PIM highlighted the importance of medication optimisation across regions to improve the health and well-being of older adults and consequently reduce health costs. 

## Data Availability

Publicly available datasets were analyzed in this study. This data can be found here: official System of Information and Monitoring of the Portuguese National Health System platform–section “how we do–como fazemos” [https://bicsp.min-saude.pt/pt/biufs/Paginas/default.aspx] (accessed on 14 July 2022).

## Supplementary Materials

The following supporting information can be downloaded at: https://www.mdpi.com/article/10.3390/ijerph191912938/s1, Table S1: Additional details of the five Administração Regional de Saúde included in the study; Table S2: Mean number of prescribed packages per 1000 inhabitants per day of top-10 level 5 ATCs, stratified by sex and age in all Portuguese regions; Table S3: Number of older adults by Portuguese regions stratified by sex and age. Table S4: Defined daily dose (DDD) per 1000 inhabitants per day in top-10 ATCs level 5, by health administrative regions (ARS) in male. Table S5: Defined daily dose (DDD) per 1000 inhabitants per day in top-10 ATCs level 5, by health administrative regions (ARS) in females.
