# Supplementary material for: Geographic Variation in Top-10 Prescribed Medicines and Potentially Inappropriate Medication in Portugal: An Ecological Study of 2.2 Million Older Adults
Source: Int J Environ Res Public Health. 2022 Oct 10;19(19):12938. doi: 10.3390/ijerph191912938 (PMC9564588; doi:10.3390/ijerph191912938)
Supplement: Supplementary file 1 [file ijerph-19-12938-s001.zip › ijerph-1926096-supplementary.pdf]

SUPPLEMENTARY MATERIAL

Table S1: Additional details of the five Administração Regional de Saúde included in the study.

<table>
<tr><th colspan="2"></th><th>North</th><th>Center</th><th>Lisbon and Tagus Valley</th><th>Alentejo</th><th>Algarve</th></tr>
<tr><td colspan="2">Gini's Coeficient, %</td><td>31.5</td><td>33.3</td><td>32.7</td><td>30.8</td><td>31.0</td></tr>
<tr><td colspan="2">Ageing Coefficient</td><td>171.8</td><td>206.8</td><td>141.2</td><td>208.9</td><td>149.2</td></tr>
<tr><td colspan="2">Ratio physicians per 1000 inhabitants</td><td>5.6</td><td>5.3</td><td>6.8</td><td>3.2</td><td>4.3</td></tr>
<tr><td rowspan="6">Educational level (65-85), %</td><td>No grade</td><td>18.1</td><td>20.0</td><td>8.1</td><td>21.7</td><td>11.3</td></tr>
<tr><td>Primary school</td><td>55.1</td><td>56.4</td><td>43.2</td><td>5.6</td><td>54.4</td></tr>
<tr><td>2nd cycle</td><td>4.9</td><td>5.2</td><td>6.7</td><td>8.7</td><td>7.1</td></tr>
<tr><td>3rd Cycle</td><td>8.6</td><td>8.8</td><td>17.7</td><td>8.7</td><td>13.6</td></tr>
<tr><td>Highschool</td><td>5.4</td><td>3.6</td><td>9.0</td><td>4.3</td><td>5.7</td></tr>
<tr><td>graduate</td><td>7.8</td><td>6.0</td><td>15.4</td><td>5.9</td><td>7.9</td></tr>
<tr><td colspan="2">Ratio urban/ rural population</td><td>5.1</td><td>1.4</td><td>149.6</td><td>1.4</td><td>2.7</td></tr>
<tr><td colspan="2">The annual amount of pension by older adult (thousand €)</td><td>5.9</td><td>5.3</td><td>7.2</td><td>5.6</td><td>6.0</td></tr>
</table>

Table S2: Mean number of prescribed packages per 1000 inhabitants per day of top-10 ATCs level 5, stratified by sex and age in all Portuguese regions.

	**Male**		**Female**		
	**65-74y**	**≥75y**	**65-74y**	**≥75y**	**Mean (SD)**
**C10AA05 Atorvastatin**	5.5	5.6	4.9	5.0	5.3 (0.4)
**A10BA02 Metformin**	3.8	3.6	3.1	2.3	3.2 (0.7)
**C07AB07 Bisoprolol**	3.0	3.8	3.0	4.1	3.5 (0.6)
**C10AA01 Simvastatin**	2.2	3.1	2.5	3.4	2.8 (0.5)
**A01AD05 Acetylsalicylic acid**	4.4	5.5	2.6	4.5	4.3 (1.2)
**A02BC02 Pantoprazole**	2.2	3.3	2.4	3.6	3.7 (1.6)
**G04CA02 Tamsulosin**	3.1	5.5			4.3 (1.7)
**N02BE01 Paracetamol**			2.1	3.4	2.7 (1.0)
**N02AJ13 Tramadol/paracetamol**			2.2	3.5	2.9 (0.9)
**C03CA01 Furosemide**		4.1		4.3	4.2 (0.1)
**M04AA01 Allopurinol**	2.0	2.8			2.4 (0.6)
**B01AC04 Clopidogrel**	1.5	2.9			2.2 (1.0)
**C10AA07 Rosuvastatin**				1.9	-
**H03AA01 Levothyroxine sodium**	2.6				-
**N05BA12 Alprazolam**			2.0		-
**A02BC01Omeprazole**			1.9		-

Legend: SD-standard deviation.

Table S3: Number of older adults by Portuguese regions stratified by sex and age.

	North	Centre	Lisbon-Tejo Valley	Alentejo	Algarv e	Total
**Male**						
65-74y	184 555	101 226	179 663	26 182	22 488	514 114
≥75y	136 395	89 265	149 744	25 023	19 431	419 858
**Female**						
65-74y	222 341	120 469	225 566	30 011	26 097	624 484
≥75y	217 495	145 101	237 389	40 659	28 990	669634
Total	760 786	456 061	792 362	121 875	97 006	2 228 090

Table S4: Defined daily dose (DDD) per 1000 inhabitants per day in top-10 ATCs level 5, by health administrative regions (ARS) in male.

Mean (SD)	**ARSN**		**ARSC**		**ARSLVT**		**ARSALE**		**ARSALG**	
C10AA05 Atorvastatin	240.3	(10.5)	403.7	(67.7)	199.6	(6.1)	157.6	(3.0)	191.3	(1.3)
A01AD05 Acetylsalicylic acid	119.6	(34.3)	275.6	(18.9)	179.0	(26.6)	108.9	(131.4)	135.0	(11.5)
G04CA02 Tamsulosin	152.2	(59.7)	248.8	(68.2)	138.7	(52.3)	114.0	(53.2)	128.3	(48.5)
A10BA02 Metformin	101.0	(8.1)	135.8	(83.0)	101.4	(5.7)	83.7	(4.4)	71.2	(2.6)
C07AB07 Bisoprolol	66.5	(10.2)	82.3	(36.3)	70.3	(9.6)	51.0	(1.7)	50.0	(2.5)
C10AA01 Simvastatin	136.0	(23.4)	163.6	(76.5)	125.5	(30.7)	113.4	(33.1)	91.5	(21.7)
A02BC02 Pantoprazole	103.7	(26.2)	139.2	(22.8)	90.0	(25.1)	132.5	(41.8)	111.9	(33.3)
M04AA01 Allopurinol	66.0	(17.3)	100.0	(2.9)	83.4	(16.9)	78.0	(11.8)	67.7	(11.5)
B01AC04 Clopidogrel	56.7	(18.1)	69.4	-	72.3	(20.6)	85.2	(30.0)	64.8	(19.6)
C03CA01 Furosemide	276.7	-	422.8	-	215.1	-	227.8	-	197.4	-

Legend: ARS - North (ARSN), Centre (ARSC), Lisbon-Tejo Valley (ARSLVT), Alentejo (ARSALE), Algarve (ARSALG).

Table S5: Defined daily dose (DDD) per 1000 inhabitants per day in top-10 ATCs level 5, by health administrative regions (ARS) in females.

Mean (SD)	**ARSN**		**ARSC**		**ARSLVT**		**ARSALE**		**ARSALG**	
C10AA05 Atorvastatin	195.0	(3.5)	202.2	(0.4)	159.7	(8.5)	144.2	(2.0)	145.2	(0.3)
C07AB07 Bisoprolol	62.0	(16.6)	53.9	(10.6)	71.3	(16.4)	60.0	(5.1)	55.4	(8.2)
A01AD05 Acetylsalicylic acid	115.6	(47.5)	101.2	(42.0)	120.8	(45.7)	139.0	(53.2)	89.6	(31.1)
C10AA01 Simvastatin	157.3	(21.4)	111.5	(21.4)	130.4	(29.6)	126.9	(31.3)	90.7	(19.9)
A10BA02 Metformin	83.5	(7.3)	65.1	(2.3)	77.8	(4.0)	73.3	-	50.8	(3.0)
N02BE01 Paracetamol	19.4	(6.7)	17.7	(6.9)	17.7	(6.7)	18.7	(6.4)	12.4	(3.5)
A02BC02 Pantoprazole	105.9	(23.9)	105.9	(30.3)	95.8	(28.5)	150.8	(42.3)	116.4	(36.4)
N02AJ13 Tramadol/paracetamol	8.4	(2.6)	9.9	(2.9)	10.6	(3.8)	10.3	(5.3)	8.4	(1.9)
C03CA01 Furosemide	304.4	-	300.2	-	217.4	-	255.7	-	192.6	-
A02BC01 Omeprazole	163.4	-		-	196.7	-	93.6	-		-

Legend: ARS - North (ARSN), Centre (ARSC), Lisbon-Tejo Valley (ARSLVT), Alentejo (ARSALE), Algarve (ARSALG); SD: standard deviation.
